# Bayesian nonparametric inference for heterogeneously mixing infectious disease models

**DOI:** 10.1073/pnas.2118425119

**Published:** 2022-03-01

**Authors:** Rowland G. Seymour, Theodore Kypraios, Philip D. O’Neill

**Affiliations:** ^a^Rights Lab, University of Nottingham, Nottingham, NG7 2RD United Kingdom;; ^b^School of Mathematical Sciences, University of Nottingham, Nottingham, NG7 2RD United Kingdom

**Keywords:** multioutput Gaussian processes, disease transmission models, foot and mouth disease, spatial epidemic models

## Abstract

Infectious disease transmission models require assumptions about how the pathogen spreads between individuals. These assumptions may be somewhat arbitrary, particularly when it comes to describing how transmission varies between individuals of different types or in different locations, and may in turn lead to incorrect conclusions or policy decisions. We develop a general Bayesian nonparametric framework for transmission modeling that removes the need to make such specific assumptions with regard to the infection process. We use multioutput Gaussian process prior distributions to model different infection rates in populations containing multiple types of individuals. Further challenges arise because the transmission process itself is unobserved, and large outbreaks can be computationally demanding to analyze. We address these issues by data augmentation and a suitable efficient approximation method. Simulation studies using synthetic data demonstrate that our framework gives accurate results. We analyze an outbreak of foot and mouth disease in the United Kingdom, quantifying the spatial transmission mechanism between farms with different combinations of livestock.

## Significance

Mathematical models of infectious disease transmission continue to play a vital role in understanding, mitigating, and preventing outbreaks. The vast majority of epidemic models in the literature are parametric, meaning that they contain inherent assumptions about how transmission occurs in a population. However, such assumptions can be lacking in appropriate biological or epidemiological justification and in consequence lead to erroneous scientific conclusions and misleading predictions. We propose a flexible Bayesian nonparametric framework that avoids the need to make strict model assumptions about the infection process and enables a far more data-driven modeling approach for inferring the mechanisms governing transmission. We use our methods to enhance our understanding of the transmission mechanisms of the 2001 UK foot and mouth disease outbreak.

The field of mathematical modeling of infectious diseases has grown significantly in the past three decades. This has led to a substantial increase in our understanding of the epidemiology and control of many diseases. The current COVID-19 pandemic has highlighted that the ability to unravel the dynamics of the spread of infectious diseases is profoundly important for designing effective control strategies, as well as assessing existing ones.

Disease spread contains inherent randomness, and capturing this aspect necessitates the use of stochastic models. The overwhelming majority of stochastic epidemic models are parametric. Such models are defined using specific probability distributions, fully specified by a finite set of parameters, which encapsulate assumptions about how transmission occurs in a population and what happens to individuals that become infected. In some cases the underlying model assumptions have biological or epidemiological justification. For example, data from case studies may suggest a suitable distribution for the time period during which individuals remain infectious (1). However, such justifications do not always exist, especially with respect to assumptions for the infection process. For example, spatial epidemic models typically assume that the transmission of the pathogen from one individual to another is a function of the distance between them, but the exact form of this function is often chosen rather arbitrarily. Nonspatial models with different types of individuals often include assumptions about how transmission potential varies with type, such as age or vaccination status. Such arbitrary assumptions can have material consequences, leading to erroneous scientific conclusions, underestimation of the uncertainty around estimates of key quantities, and misleading predictions (2).

An alternative to parametric epidemic modeling is to adopt a nonparametric approach in which the specific finite-parameter probability distributions in parametric models are replaced by infinite-parameter versions. This avoids having to make particular model assumptions and enables the modeling exercise to be far more data driven. Although general nonparametric statistical theory has a long history, there has been relatively little work to adapt the ideas to epidemic modeling. To date, most attention has been directed toward estimation of how infection rates vary over time, in both classical (3, 4) and Bayesian (5–7) statistical frameworks.

In this paper we develop nonparametric stochastic epidemic models that allow transmission potential to vary between individuals. This is a wide class of models that include spatial models, multitype models, and models on static networks. Fitting such models to data is a nontrivial exercise, due to the facts that the transmission process itself is unobserved in reality and that the models are inherently infinite dimensional. We develop computational methods for fitting the models to data in a Bayesian statistical framework, making use of data augmentation Markov chain Monte Carlo (MCMC) methods and suitable approximations.

We use our methods to enhance understanding of the mechanisms of foot and mouth disease (FMD) transmission. Disease among livestock can cause severe economic consequences to the agriculture industry, concern to consumers, and the culling of millions of animals. In the 2001 FMD outbreak in the United Kingdom, over 6 million animals were culled with a cost to the public and private purse of over £8 billion (8). Numerous studies have used parametric epidemic models to analyze data on disease outbreaks among livestock (9–18), often with a view to understanding the spatial spread of disease, determining factors that affect the potential infectivity or susceptibility of farms, and assessing existing or proposed control measures. Our approach dispenses with the need for the underlying transmission assumptions of parametric models, instead allowing the analysis to be driven by evidence in the data.

## Methods

### Epidemic Model.

We now describe an epidemic model that generalizes the classical continuous-time susceptible–infective–removed (SIR) model (19). Consider a closed population containing *N* individuals labeled 1,…,N. Each individual *j* has a set of covariates $\phi_{j}$, such as location or type, which remains unchanged throughout the epidemic.

At any time, each individual is susceptible to the disease, infected with the disease and infective, or removed, meaning that they have had the disease but are now unable to infect others. In practice, removal may refer to isolation, natural recovery and immunity, or death, depending on the pathogen being modeled. Removed individuals cannot be reinfected. Initially, the population is entirely susceptible other than a few infectives. Infective individuals remain so for a time period drawn from some specified nonnegative probability distribution, after which they enter the removed class. The infectious periods of different individuals are assumed to be mutually independent.

During the infectious period, an infective individual *i* has contacts with any given susceptible individual *j* in the population at times given by the points of a Poisson process of rate ${\tilde{\beta }}_{ij}$. If a contact occurs, then *j* immediately becomes infective. The Poisson processes corresponding to different pairs of individuals are assumed to be mutually independent. We assume that ${\tilde{\beta }}_{ij}=\beta_{ij}(\phi_{i},\phi_{j})$ for some function *β_ij_*. The epidemic ends when there are no more infectives remaining.

### Nonparametric Modeling.

The overwhelming majority of epidemic models of the kind just described specify the infection rate functions *β_ij_* explicitly by assuming a particular parametric form. Conversely, in this paper we attempt to estimate such functions nonparametrically in a Bayesian framework. Technically, this involves assigning prior distributions to the set of possible *β_ij_* functions and then using an MCMC algorithm to sample from the resulting posterior distributions, given observed data from an epidemic outbreak.

For the remainder of this paper we focus on multitype susceptibility models (20) in which individuals can have varying susceptibility to the disease, but are assumed to be equally infectious if infected. Specifically, we assume that each individual is one of a possible *p* types labeled 1,…,p and that $\beta_{ij}=\beta^{\left(k\right)}$ if *j* is type *k*, k=1,…,p. However, our methods can equally be applied in a more general setting.

### Multioutput Gaussian Processes.

To fit the epidemic model to data in a Bayesian framework, we must assign a prior distribution to the vector of functions $\left(\beta^{\left(1\right)},\ldots ,\beta^{\left(p\right)}\right)$, which can be naturally achieved by using multioutput Gaussian processes (GPs). Recall that if a real-valued function *f* has a GP distribution, then for any vector $\left(x_{1},\ldots ,x_{n}\right)$ of values in the domain of *f*, $\left(f(x_{1}),\ldots ,f(x_{n})\right)$ has a multivariate normal distribution specified by its mean function, *μ*, and positive definite covariance matrix function Σ, where

$$\begin{array}{ll} \mu (x_{i}) & =E[f(x_{i})],\\ \Sigma_{i,j}(x_{i},x_{j}) & =E[(f(x_{i})-\mu (x_{i}))(f(x_{j})-\mu (x_{j}))], \end{array}$$

and we denote this by f∼GP(μ,Σ).

Although our methodology applies to any choice of Σ, we henceforth focus on the squared exponential covariance function k(·,·) in which *f* has domain R and

$$\begin{array}{ll} \Sigma_{i,j}(x_{i},x_{j}) & =k(x_{i},x_{j};\alpha ,l),\\ k(x_{i},x_{j};\alpha ,l) & =\alpha^{2}\exp\left\{-\frac{{\left(x_{i}-x_{j}\right)}^{2}}{l^{2}}\right\}, \end{array}$$

where *α* and *l* are the hyperparameters of the GP, known respectively as the variance and the length scale. Multioutput GPs extend these ideas in a natural way to multiple functions $f^{\left(1\right)},\ldots ,f^{\left(p\right)}$ by introducing covariance between the functions.

In our setting, each input value *x_k_* will be a real-valued function of a covariate pair $\left(\phi_{i},\phi_{j}\right)$, for example, the distance between *i* and *j*. As functions with GP distributions are real valued we use a nonnegative function *g*, typically g=exp, to transform samples from the GP into nonnegative infection rate functions by defining

$$\beta^{\left(j\right)}=g\left(f^{\left(j\right)}\right),\;j=1\ldots ,p.$$

We now use this approach to define three different models.

#### The multioutput covariance model.

For the multioutput covariance (MOC) model, we place a joint GP prior distribution on the functions $f^{\left(1\right)},\ldots f^{\left(p\right)}$. Specifically, we assume that

$$\left(\begin{array}{c} f^{\left(1\right)}\\ f^{\left(2\right)}\\ \vdots \\ f^{\left(p\right)} \end{array}\right)∼GP\left(0,\;\left(\begin{array}{ccc} \Sigma^{\left(1,1\right)} & \cdots & \rho_{1,p}\Sigma^{\left(1,p\right)}\\ \rho_{2,1}\Sigma^{\left(2,1\right)} & \cdots & \rho_{2,p}\Sigma^{\left(2,p\right)}\\ \vdots & & \vdots \\ \rho_{p,1}\Sigma^{\left(p,1\right)} & \cdots & \Sigma^{\left(p,p\right)} \end{array}\right)\right),$$

so that for any input vector $\left(x_{1},\ldots ,x_{n}\right)$, where $x_{i}=(x_{i}^{\left(1\right)},\ldots ,$ $x_{i}^{\left(p\right)}),\;\Sigma_{i,j}^{\left(a,b\right)}(x_{i}^{\left(a\right)},x_{j}^{\left(b\right)})=k(x_{i}^{\left(a\right)},x_{j}^{\left(b\right)};\alpha ,l)$ and $\rho_{j,k}$ is a measure of the correlation between $f^{\left(j\right)}$ and $f^{\left(k\right)}$ satisfying $-1\leq \rho_{j,k}\leq 1$ and $\rho_{j,k}=\rho_{k,j}$ for k≠j. Note that we assume all covariance functions have the same length-scale hyperparameter; this is not necessary, but in practical applications of the kind we consider, data are typically insufficient to estimate numerous length-scale parameters.

#### The independent GP model.

Setting $\rho_{j,k}=0$ for all *j* and *k* gives rise to an independent GP (IGP) model for which it is assumed that there is no relationship between the infection rates acting on different types of individuals a priori. An advantage of this model is its simplicity, because we do not have to specify the relationship between $f^{\left(j\right)}$ and $f^{\left(k\right)}$. We may also allow the *p* independent GPs to have their own length scales.

#### The discrepancy-based model.

In the discrepancy-based (DB) model we first set $f^{\left(1\right)}$ as a baseline, to which we assign a GP prior with mean zero and covariance matrix $\Sigma_{j,k}^{\left(1\right)}$. For j=2,…p we then assume that

$$f^{\left(j\right)}=f^{\left(1\right)}+u^{\left(j\right)},\;u^{\left(j\right)}∼GP\left(0,\;\Sigma_{j,k}^{\left(j\right)}\right),$$

where $u^{\left(j\right)}$ represents the discrepancy between $f^{\left(j\right)}$ and $f^{\left(1\right)}$, with $f^{\left(1\right)},u^{\left(2\right)},\ldots ,u^{\left(p\right)}$ assumed to be mutually independent. We further assume that $\Sigma_{j,k}^{\left(j\right)}(x_{j},x_{k})=k(x_{j},x_{k};\;\alpha ,l_{j})$, for j=1,…p, so that in particular the discrepancies have individual length scales. When fitted to data, this model enables a direct comparison between infection rates of different types of individuals to be made, which can be useful for policy makers.

### Data and Likelihood Function.

Consider an outbreak of disease among a population of *N* individuals, *n* of which were infected. We assume that we observe the removal times of the *n* infected individuals, but not their infection times. In practice, the likelihood of the observed removal times under our model is analytically and computationally intractable. This is because the calculation involves integrating over the unobserved infection times, which lie in a nontrivial subset of $R^{n}$. Following ref. 21 we proceed by introducing the unobserved infection times in a data-augmentation framework, which in turn yields a tractable data-augmented likelihood.

Label the infected individuals 1,…,n by their removal time and the remaining individuals n+1,…,N arbitrarily. We denote the infection and removal time of individual *j* by *i_j_* and *r_j_*, respectively, and assume that the epidemic starts with a single infective individual labeled *ω*. Define $i=\{i_{1},\ldots ,i_{\omega -1},i_{\omega +1},\ldots ,i_{N}\}$ to be the set of infection times excluding the initial infection time $i_{\omega }$ and $r=\{r_{1},\ldots ,r_{N}\}$ to be the set of removal times where $r_{1}<r_{2}<\ldots <r_{N}$. If an individual *j* has not been infected, we set $i_{j}=r_{j}=\infty$. We assume the population consists of p≪N types of individuals labeled 1,…,p and define *c_j_* to be the type of individual *j*.

In the following, we assume that infectious periods follow a Gamma distribution, although our methods can easily be adapted for any other choice. We also assume that the infection rate from individual *j* to individual *k* is $\beta^{\left(c_{k}\right)}(x_{j,k})$, where $x_{j,k}=D(\phi_{j},\phi_{k})\geq 0$ for some specified function *D*. In practice, *D* will be some measure of distance between individuals *j* and *k*. We then have the data-augmented likelihood function

$$\begin{array}{ll} \pi & \left(i,r|\beta^{\left(1\right)},\ldots ,\beta^{\left(p\right)},\lambda ,\gamma ,\omega ,i_{\omega })=\prod_{j=1}^{n}h(r_{j}-i_{j}|\lambda ,\gamma \right)\\ & \times \prod_{\begin{array}{c} j=1\\ j\neq \omega \end{array}}^{n}\left(\sum_{k\in Y_{j}}\beta^{\left(c_{j}\right)}(x_{k,j})\right)\exp\left\{-\sum_{j=1}^{n}\sum_{k=1}^{N}\beta^{\left(c_{k}\right)}(x_{j,k})\delta_{j,k}\right\}, \end{array}$$

where h(·|λ,γ) denotes the probability density function of a Gamma distribution with shape and rate parameters *λ* and *γ*, respectively; $Y_{j}$ denotes the set of individuals who are infective at time *i_j_*, excluding *j*; and $\delta_{j,k}=\min(r_{j},i_{k})-\min(i_{j},i_{k})$.

The likelihood function consists of three parts. The first part is the likelihood of the infectious periods of all infected individuals. The second part accounts for individuals becoming infected, and the third part is the probability of individuals avoiding infection throughout the epidemic. Note that $\delta_{j,k}$ is the time during which individual *k* avoids infection from individual *j*.

### Bayesian Inference and Prior Distributions.

Since it is typically difficult to accurately estimate both the shape and rate parameters of a Gamma infectious period distribution given data on removals alone, we follow ref. 12 and treat the shape parameter *λ* as fixed and known. In our applications, the GP variance hyperparameter *α* can also be hard to estimate and so this is also assumed to be known. Our main objective is then to estimate the infection rate functions $\beta =(\beta^{\left(1\right)},\ldots ,\beta^{\left(p\right)})$, which are specified by the corresponding GP length-scale hyperparameters $l_{1},\ldots ,l_{m}$ (where *m* = 1 or *m* = *p* depending on the choice of GP prior model) and, for the MOC model, the correlation parameters $\rho =\left\{\rho_{j,k}\right\}$. We also estimate the infectious period distribution rate parameter *γ*, the unobserved infection times, and the parameters relating to the initial infected individual, *ω* and $i_{\omega }$. By assigning mutually independent prior distributions in the natural manner, the posterior density is specified by

[1] $$\begin{array}{ll} & \pi (\beta ,l_{1},\ldots ,l_{m},\rho ,\gamma ,i,\omega ,i_{\omega }|r,\lambda )\\ & \propto \;\pi (i,r|\beta ,\lambda ,\gamma ,\omega ,i_{\omega })\pi (\beta |l_{1},\ldots ,l_{m},\rho )\\ & \times \pi (l_{1})\cdots \pi (l_{m})\pi (\rho )\pi (\gamma )\pi (\omega )\pi (i_{\omega }|\omega ). \end{array}$$

Let Exp(*a*) denote an exponential random variable with mean $a^{-1}$. We assume a priori that $l_{j}∼\text{Exp}(\chi_{l_{j}}),\;\gamma ∼\text{Exp}(\chi_{\gamma })$, that *ω* is uniformly distributed on {1,…,n}, and that $r_{1}-i_{\omega }∼\text{Exp}(\chi_{\omega })$. For the MOC model, we assume that the $\rho_{j,k}$ are independently uniformly distributed on [−1,1] a priori. Further details can be found in *SI Appendix*.

### Posterior Computation via MCMC.

We use a bespoke data-augmentation MCMC algorithm to sample from the posterior distribution, an outline of which is shown in *Algorithm 1* and in which step 3 is necessary only if the MOC model is used. Details are given in *SI Appendix*.

Algorithm 1 Basic structure of the MCMC algorithms:

1) Initialize the chain with values $\gamma^{\left(0\right)},\;\beta^{\left(0\right)},\;l_{1}^{\left(0\right)},\ldots ,l_{m}^{\left(0\right)},\;\rho^{\left(0\right)},\;i^{\left(0\right)},\;\omega^{\left(0\right)}$, and $i_{\omega }^{\left(0\right)}$.
  Repeat the following steps:
2) Update *β* using a Metropolis–Hastings step;
3) Update ρ using a Metropolis–Hastings step;
4) Update GP hyperparameters using a Metropolis–Hasting step;
5) Update *γ* using a Gibbs step;
6) Update *ω* and an infection time $i_{\omega }|\omega$ using a Metropolis–Hastings step;
7) Choose an infection time at random and update it using a Metropolis–Hastings step.

### Mean Projection Approximation.

Computing the term $\pi (\beta |l_{1},\ldots ,$ $l_{m},\rho )$ in Eq. **1** requires evaluation of the probability density function of a multivariate normal distribution, which in turn requires computing the inverse of its covariance matrix. This can be computationally demanding in high dimensions (22–24); in our setting, we found population sizes of more than about *N* = 300 individuals to be problematic. To resolve this issue we used the mean projection approximation (MPA). MPA essentially works by using a subset of the original dataset that is suitably representative of the original one (e.g., its size is sufficiently large and its elements are suitably placed across the entire domain to capture the features of *β*), inferring the infection rate functions given this subset, and then projecting the result onto the full dataset to obtain *β*. Full details are given in *SI Appendix*.

## Results

We demonstrate our methods using simulated and real data. Our focus is the spread of disease in livestock settings, where individuals in the epidemic model correspond to farms in some geographic region. Code to reproduce this analysis in R and C is available at github.com/rowlandseymour/BNP_4_HMSEM.

### Synthetic Data for Two Types.

We carried out a simulation study to test our methods in the setting of multiple types of individuals. The locations of 1,000 farms were randomly generated on a unit square, half being type 0, and half type 1. We simulated 250 epidemic outbreaks using the infection rates

[2] $${\tilde{\beta }}_{ij}=\{\begin{array}{ll} \beta^{\left(0\right)}(d_{ij})=\beta_{0}\exp\{-3d_{ij}\} & \text{if}\;\text{farm}\;j\;\text{is}\;\text{type}\;0\\ \beta^{\left(1\right)}(d_{ij})=\beta_{1}\exp\{-2d_{ij}\} & \text{if}\;\text{farm}\;j\;\text{is}\;\text{type}\;1, \end{array}$$

where *d_ij_* denotes the Euclidean distance between farms *i* and *j*, with $\beta_{0}=0.005,\;\beta_{1}=0.001$, and γ=3. We used our methods to infer the model parameters for each dataset, with fixed GP hyperparameters *α* = 6 for all models and length scales *l* = 5 for the MOC and IGP models. The latter was done because we encountered some numerical instabilities when trying to estimate *l* separately.

**Fig. 1.**
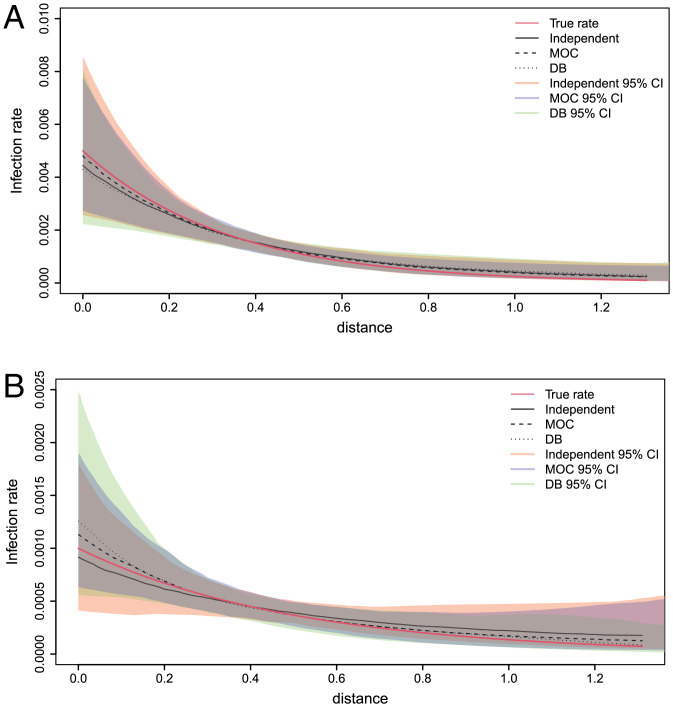
Synthetic data: Median estimates of the infection rate functions under each model compared to the true infection rate function. (*A*) Estimates for the type 0 infection rate. (*B*) Estimates for the type 1 infection rate.

The results for the infection rate functions are shown in Fig. 1 and Table 1. Broadly speaking, all three models are able to successfully estimate the true infection rate functions given the available data. There is more uncertainty for the estimation of the type 1 infection rate function $\beta^{\left(1\right)}$, which is to be expected since $\beta^{\left(1\right)}(d)$ is considerably less than $\beta^{\left(0\right)}(d)$ for typical *d* in the simulated datasets, and hence fewer type 1 farms get infected. To assess the results for the infection times, we use the relative error in the sum of the infection times. This is defined, for a single simulated dataset, as $\bar{i}=(S-\hat{S})/S$, where *S* denotes the true sum of infection times of infected farms and $\hat{S}$ is its median estimate from the MCMC output. As shown in Table 1, the relative error for all methods is small, which demonstrates that our method for inferring infection times gives accurate results. More numerical comparisons are given in *SI Appendix*, Table S1.

**Table 1.** Medians and 95% credible intervals for the model parameters using the three models, compared to the true model parameters

Model	Parameter	Study median	95% credible interval
IGP	*β*_0_	0.00446	(0.00257, 0.00859)
	*β*_1_	0.000920	(0.00415, 0.00180)
	*γ*	3.13	(2.41, 3.92)
	$\tilde{i}$	-0.0111	(–0.0791, 0.0470)
MOC	*β*_0_	0.00484	(0.00273, 0.00782)
	*β*_1_	0.00113	(0.000644, 0.00191)
	*γ*	3.07	(2.39, 3.89)
	$\tilde{i}$	-0.00757	(–0.0839, 0.0514)
	*ρ*	0.762	(0.495, 0.856)
DB	*β*_0_	0.00430	(0.00223, 0.0808)
	*β*_1_	0.00126	(0.000562, 0.00250)
	*γ*	3.11	(2.43, 4.02)
	$\tilde{i}$	-0.00989	(–0.102, 0.0505)
	*l*_1_	5.05	(2.49, 10.7)
	*l*_2_	6.87	(2.14, 16.3)

### Foot and Mouth Disease.

In 2001 there was a large outbreak of FMD in sheep and cattle farms in the United Kingdom, resulting in over 2,000 cases of disease and the slaughter of over 6 million animals. In the county of Cumbria, which was the most affected area, there were 5,436 farms consisting of $N_{1}=1,061$ sheep farms, $N_{2}=1,064$ cattle farms, and $N_{3}=3,253$ farms with both sheep and cattle. Of these farms, *n* = 1, 021 were infected including 8% of sheep farms, 13% of cattle farms, and 24% of farms where both sheep and cattle were present. We focus on the Cumbria data.

The 2001 UK FMD outbreak has been studied extensively in the modeling literature (9, 11, 12, 18, 25) with a particular focus on proposing and fitting models where the infection rate between farms is assumed to depend on the Euclidean distance between them, as well as the number of the different types of animals on each farm. However, the proposed models have strict parametric assumptions with regard to the functional form of the spatial dependency and the effect of the numbers of animals of different types in each farm. Given that such models are often used during the course of an outbreak to inform policy making, it is important to consider data-driven alternatives such as the Bayesian nonparametric approach described above, which avoids the need to make arbitrary assumptions about infection rate functions.

We split the farms into three types: sheep farms, cattle farms, and farms with both sheep and cattle. As the number farms of each type differs considerably, we standardize the rates by the number of farms of that type by defining

$${\tilde{\beta }}_{jk}=\{\begin{array}{l} \frac{1}{N_{1}}\exp\left(f^{\left(1\right)}(d_{j,k})\right)\;\text{if}\;k\;\text{is}\;\text{a}\;\text{sheep}\text{-}\text{only}\;\text{farm,}\\ \frac{1}{N_{2}}\exp\left(f^{\left(2\right)}(d_{j,k})\right)\;\text{if}\;k\;\text{is}\;\text{a}\;\text{cattle}\text{-}\text{only}\;\text{farm,}\\ \frac{1}{N_{3}}\exp\left(f^{\left(3\right)}(d_{j,k})\right)\;\text{if}\;k\;\text{has}\;\text{sheep}\;\text{and}\;\text{cattle}, \end{array}$$

where the *f* functions are assigned GP prior distributions as described above and where $d_{j,k}$ denotes the distance between farms *j* and *k*. We set GP hyperparameters as *α* = 6 and *l* = 8.5 for all models, motivated by the results of a simpler analysis described in ref. 26. We ran all MCMC algorithms for 25,000 iterations, discarding the first 5,000 as a burn-in period. This took around 1 d to complete using the University of Nottingham High Performance Computing Service.

#### MOC model.

**Fig. 2.**
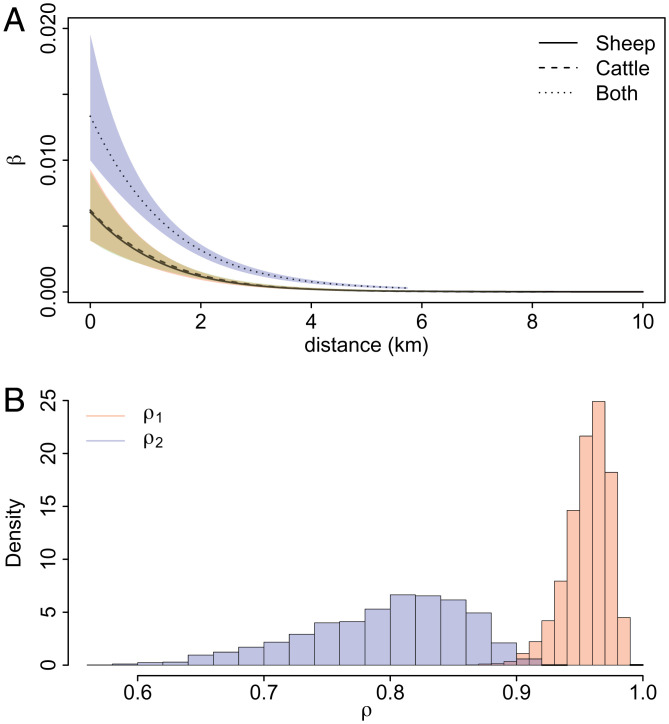
Results of the MOC model applied to the FMD dataset. (*A*) Posterior medians and 95% credible intervals for the infection rate functions. (*B*) The posterior distributions for the correlation parameters *ρ*_1_ and *ρ*_2_.

We used the MOC model with two correlation parameters, assuming the correlation between the sheep-only and sheep-and-cattle farms is the same as the correlation between the cattle-only and sheep-and-cattle farms. The results in Fig. 2 show that farms with both sheep and cattle are more susceptible to contracting the disease than farms with only one type of animal. With regard to the shape of the infection rate functions, the function for sheep-and-cattle farms decays more quickly than the other two functions, and for farms of all types the probability of an infected farm infecting a susceptible farm farther than 7 km away is negligible.

Fig. 2*A* shows strong similarity between the infection rate functions for sheep-only and cattle-only farms. Fig. 2*B* shows the correlation between these two functions is high and the 95% credible interval is (0.914, 0.982). The correlation between the functions for farms with one type of animal and for farms with both types of animals is not as high, but still indicates considerable positive correlation [95% CI: (0.652, 0.891)]. The posterior median for the infectious period distribution rate parameter *γ* is 0.508, which gives an expected infectious period of 7.86 d. This is in line with estimates reported in refs. 12 and 27, namely 7.55 and 7.69 d, respectively, obtained using parametric methods.

#### Discrepancy-based model.

**Fig. 3.**
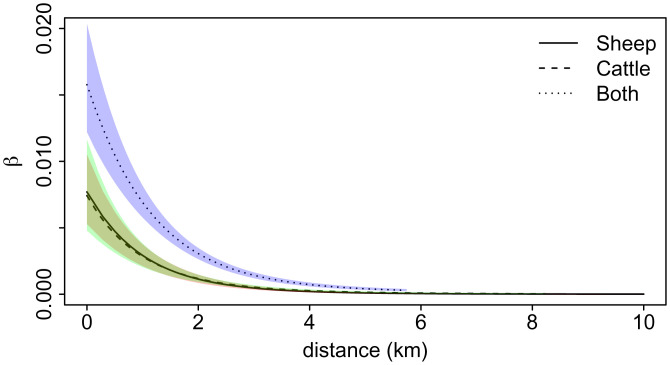
Results of the DB model applied to the FMD dataset: Posterior medians and 95% credible intervals for the infection rate functions.

The results are shown in Fig. 3 and are similar to those for the MOC model. In contrast to the MOC model, we can compare the functions to a baseline, chosen to be the infection rate function for sheep-only farms. Fig. 3 shows that there is little difference for cattle-only farms, but that the infection rate function for sheep-and-cattle farms is significantly higher than the sheep-only infection rate function for distances less than around 3 km. The posterior median for *γ* is 0.517 [95% CI:(0.469, 0.570)], which gives an average infectious period of 7.74 d.

### Assessing Disease Control Strategies.

We implemented a further simulation study, the details of which are given in *SI Appendix*, section 4A, to demonstrate the benefits of our Bayesian nonparametric framework by directly comparing it to a parametric approach. We chose 1,000 farms uniformly at random from the 2001 UK FMD data and simulated an outbreak assuming that all farms were of the same type and with infection rate ${\tilde{\beta }}_{ij}=\beta_{ij}(d_{ij})=0.3\times 0.0015{\left(1+{\left(d_{ij}-2\right)}^{2}\right)}^{-1}+0.7\times 0.0015$ ${\left(1+d_{ij}\right)}^{-1}$, where *d_ij_* denotes the Euclidean distance between farms *i* and *j*. This infection rate is a weighted mixture of two parametric functions: a logistic and a heavy-tailed Cauchy function with the latter allowing for long-range transmission (12). Infectious periods were assumed to be Gamma distributed with mean 6 d and SD 3.46 d. The simulated outbreak lasted 57 d and 782 farms were infected. We fitted six models, the only difference between them being the assumption about the functional form of the infection rate function (Table 2). We fixed the infectious period distribution shape parameter as λ=3 and inferred the rate parameter *γ*, assuming γ∼Exp(0.01) a priori. The results show that only the Bayesian nonparametric model (*M*_6_) can detect the mixture nature of the true infection rate (*SI Appendix*, Fig. S1). This feature has important practical implications in terms of implementing control measures, because prevention of short-range infections is typically achieved by different means from those required to prevent long-range infections.

**Table 2.** Assessing disease control strategies: Results of the ring-culling strategy and time taken to run the MCMC algorithm

Model	Infection function ($\beta_{ij})$	Mean final size	Severe outbreak probability	Time, min
*M*_1_	$0.3\times \frac{\theta_{1}}{\theta_{2}+{\left(d_{ij}-\theta_{3}\right)}^{2}}+$	370	0.634	10
	$0.7\times \frac{\theta_{1}}{\theta_{4}+d_{ij}}$			
*M*_2_	$\frac{\lambda_{1}}{\lambda_{2}+d_{ij}}$	575	0.609	2
*M*_3_	$\nu_{1}\exp(-\nu_{2}d_{ij})$	402	0.450	2
*M*_4_	$\frac{\sigma_{1}}{\sigma_{2}+d_{ij}^{2}}$	274	0.645	2
*M*_5_	$\frac{\psi_{1}}{\psi_{2}+{\left(d_{ij}-\psi_{3}\right)}^{2}}$	391	0.511	5
*M*_6_	$\exp(f(d_{ij}))$	362	0.590	60

Following ref. 28, we investigated the predicted efficacy of a ring-culling strategy as a disease control measure, full details of which can be found in *SI Appendix*. The results in Table 2 show the resulting predicted mean final size and probability of a severe outbreak, the latter defined as one in which 10% of farms were infected. Model *M*_1_ is the true model, and models *M*_2_ and *M*_4_ estimate the probability of a severe outbreak fairly well but fail to predict the correct final size. Model *M*_2_ correctly estimates the infection rate over short distances, but the infection rate function decays more slowly than that in the true model. Models *M*_3_ and *M*_5_ have better estimates of final size but do not estimate the outbreak probability well. The existence of long-range transmission in the simulated data causes the decay rate parameter in model *M*_3_ to be underestimated, and in consequence the infection rate over short distances in the model is an order of magnitude smaller than in the true model. Thus, using models *M*_2_ to *M*_4_ for planning purposes would inevitably lead to misleading conclusions. Conversely, our nonparametric approach matches the results from the true model *M*_1_ for both mean final size and the probability of a severe outbreak and does so without having to specify the parametric form of the infection rate function. An additional simulation study that further demonstrates the benefit of our approach is given in *SI Appendix*, section 4B.

## Discussion

We have presented a framework for Bayesian nonparametric inference for infection rate functions in individual-level stochastic epidemic models. Although motivated by models for livestock diseases, the methodology is applicable to a wide class of epidemic models, including household models, network models, and age-structured models. The key benefit of our approach is that it removes the need to make specific parametric assumptions about infection rate functions. Instead, we need only make more general assumptions, such as the smoothness of the function we wish to infer. We have also demonstrated that our approach can be used successfully for large datasets by employing MPA methods.

Our methods are based on multioutput GPs, which allows us to incorporate a priori beliefs that there is a shared structure between the infection rates for individuals of different types. The multioutput covariance model assumes the infection rates for individual types are correlated, whereas the discrepancy-based model enables the infection rate for each type to be compared to a baseline infection rate. The independent GP model is a simpler model to which we can compare the MOC and DB models, which assumes that the infection rate functions for different types are mutually independent.

A key practical difference between the MOC and DB models is the intended audience. From a mathematical viewpoint, being able to characterize the covariance between two functions is useful and the MOC framework allows us to do this. It also allows us to describe the relationship between two types with one correlation parameter, *ρ*. However, such information may be less interpretable to practitioners than direct comparisons between infection rate functions, as provided by the DB model.

Our methods can be computationally intensive in practice. Updating the length-scale parameter is a bottleneck in the MCMC algorithm as this step involves decomposing and inverting a covariance matrix, and there is also considerable correlation between the infection rate function and length-scale parameter samples. Issues can also arise via the data-augmentation MCMC scheme due to inherent correlations between the unobserved infection times and the model parameters. There are various potential approaches to dealing with these computational difficulties, one of which is to use the approximate-likelihood method described in ref. 29 to remove the need for data augmentation. This in turn would increase the utility of our methods for real-time inference during an outbreak. Furthermore, it would also be of interest to demonstrate the utility of our modeling framework in different contexts beyond spatial epidemic models, such as static network diffusion processes (30).

## Supplementary Material

Supplementary File

## Data Availability

Code and synthetic data have been deposited in Github (https://github.com/rowlandseymour/BNP_4_HMSEM). Great Britain’s (GB) farm demography data is available at a national level by contacting GB’s Animal and Plant Health Agency at enquiries@apha.gov.uk.
