# Supplementary material for: Bayesian nonparametric inference for heterogeneously mixing infectious disease models
Source: Proc Natl Acad Sci U S A. 2022 Mar 1;119(10):e2118425119. doi: 10.1073/pnas.2118425119 (PMC8915959; doi:10.1073/pnas.2118425119)
Supplement: Supplementary File [file pnas.2118425119.sapp.pdf]

Supplementary Information for

Bayesian nonparametric inference for heterogeneously-mixing infectious disease models

R. G. Seymour, T. Kypraios, P. D. O'Neill

Theodore Kypraios.
E-mail: theodore.kypraios@nottingham.ac.uk

This PDF file includes:

Supplementary text
Figs. S1 to S2 (not allowed for Brief Reports)
Tables S1 to S2 (not allowed for Brief Reports)
SI References

Supporting Information Text

1. Posterior Computation via MCMC

We adopt a data-augmentation framework in which the unobserved infection times are treated as additional unknown parameters. The data-augmented likelihood is given by:

$$\pi(\mathbf{i}, \mathbf{r} | \beta^{(1)}, \ldots, \beta^{(p)}, \lambda, \gamma, \omega, i_\omega) = \prod_{j=1}^{n} h(r_j - i_j | \lambda, \gamma) \times \prod_{\substack{j=1 \\ j \neq \omega}}^{n} \left(\sum_{k \in \mathcal{Y}_j} \beta^{(c_j)}(x_{k,j}) \right) \exp\left\{ - \sum_{j=1}^{n} \sum_{k=1}^{N} \beta^{(c_k)}(x_{j,k}) \delta_{j,k} \right\},$$

where $h(\cdot|\lambda, \gamma)$ denotes the probability density function of a Gamma distribution with shape and rate parameters λ and γ, respectively; $\mathcal{Y}_j$ denotes the set of individuals who are infective at time i_j, excluding j; c_j is the type of individual j and ϕ_j is a set of their covariates; $x_{j,k} = D(\phi_j, \phi_k) \geq 0$ for some specified function D and $\delta_{j,k} = \min(r_j, i_k) - \min(i_j, i_k)$. The general form of the posterior density up to proportionality is given as follows:

$$\pi(\beta, l_1, \ldots, l_m, \boldsymbol{\rho}, \gamma, \mathbf{i}, \omega, i_\omega | \mathbf{r}, \lambda) \propto \pi(\mathbf{i}, \mathbf{r} | \beta, \lambda, \gamma, \omega, i_\omega)\, \pi(\beta | l_1, \ldots, l_m, \boldsymbol{\rho})\, \pi(l_1) \cdots \pi(l_m)\, \pi(\boldsymbol{\rho})\, \pi(\gamma)\, \pi(\omega)\, \pi(i_\omega | \omega),$$

where $\beta = \left(\beta^{(1)}, \ldots, \beta^{(m)}\right)$. We use a bespoke data-augmentation MCMC algorithm, an outline of which is given below:

Algorithm 1 Basic Structure of the MCMC Algorithms

1: Initialize the chain with values $\gamma^{(0)}$, $\beta^{(0)}$, $l_1^{(0)}, \ldots, l_m^{(0)}$, $\boldsymbol{\rho}^{(0)}$, $\mathbf{i}^{(0)}$, $\omega^{(0)}$ and $i_\omega^{(0)}$
Repeat the following steps
2: Update β using a Metropolis-Hastings step;
3: Update $\boldsymbol{\rho}$ using a Metropolis-Hastings step;
4: Update GP hyperparameters using a Metropolis-Hasting step;
5: Update γ using a Gibbs step;
6: Update ω and an infection time $i_\omega|\omega$ using a Metropolis-Hastings step;
7: Choose an infection time at random and update it using a Metropolis-Hastings step.

The details on how the infection rate function β and the corresponding GP hyperparameters are updated (Steps 2 and 3) differ between the three different models i) Multi-Output Covariance (MOC), ii) Independent GP (IGP) or iii) Discrepancy-Based (DB) which we describe in detail below, along with the details on how to implement Steps 4-6.

A. Updating Parameters Specific to the MOC Model. For the MOC model we assume that all covariance functions have the same length scale parameter, i.e. $l_1 = \ldots l_m = l$ and assign an Exponential prior distribution to l with mean $1/\chi_l$. Furthermore, the correlation parameters $\boldsymbol{\rho} = \{\rho_{j,k}\}$, for $j = 1, \ldots, p$, $k = 1, \ldots, p$ with $j < k$, are assumed to be independently uniformly distributed on $[-1, 1]$ *a priori*. Hence, the target posterior density up to proportionality is given by:

$$\begin{aligned} \pi(\beta, l, \boldsymbol{\rho}, \gamma, \mathbf{i}, \omega, i_\omega | \mathbf{r}, \lambda) &\propto \pi(\mathbf{i}, \mathbf{r} | \beta, \lambda, \gamma, \omega, i_\omega)\, \pi(\beta | l, \boldsymbol{\rho})\, \pi(l)\, \pi(\boldsymbol{\rho})\, \pi(\gamma)\, \pi(\omega)\, \pi(i_\omega | \omega) \\ &\propto \prod_{j=1}^{n} h(r_j - i_j | \lambda, \gamma) \prod_{\substack{j=1 \\ j \neq \omega}}^{n} \left(\sum_{k \in \mathcal{Y}_j} \beta^{(c_j)}(x_{k,j}) \right) \exp\left\{ - \sum_{j=1}^{n} \sum_{k=1}^{N} \beta^{(c_k)}(x_{j,k}) \delta_{j,k} \right\} \\ &\times \mathcal{GP}(\log \beta; 0, \Sigma) \exp\{\chi_\omega \omega\} \exp\{-\chi_\gamma \gamma\} \exp\{-\chi_l l\}, \end{aligned}$$

where

$$\Sigma = \begin{pmatrix} \Sigma^{(1,1)} & \cdots & \rho_{1,p}\Sigma^{(1,p)} \\ \rho_{2,1}\Sigma^{(2,1)} & \cdots & \rho_{2,p}\Sigma^{(2,p)} \\ \vdots & & \vdots \\ \rho_{p,1}\Sigma^{(p,1)} & \cdots & \Sigma^{(p,p)} \end{pmatrix}. \qquad [1]$$

Updating the infection rate functions $\beta = \left(\beta^{(1)}, \ldots, \beta^{(p)}\right)$: We update the infection rate functions for all types in a block, using the underrelaxed proposal mechanism (1) which allows us to efficiently propose values for each infection rate function $\beta^{(j)} = \exp(f^{(j)})$ across the whole domain.

Step 2 of Algorithm 1 for the MOC Model

Draw $\nu^{(1)}, \dots, \nu^{(p)}$ from the joint GP prior distribution:

$$\begin{pmatrix} \nu^{(1)} \\ \nu^{(2)} \\ \vdots \\ \nu^{(p)} \end{pmatrix} \sim \mathcal{GP}\left(0, \begin{pmatrix} \Sigma^{(1,1)} & \cdots & \rho_{1,p}\Sigma^{(1,p)} \\ \rho_{2,1}\Sigma^{(2,1)} & \cdots & \rho_{2,p}\Sigma^{(2,p)} \\ \vdots & & \vdots \\ \rho_{p,1}\Sigma^{(p,1)} & \cdots & \Sigma^{(p,p)} \end{pmatrix}\right);$$

for $j = 1, \dots p$ **do**

  Propose a new function $f^{(j)'} = \sqrt{1-\delta^2} f^{(j)} + \delta \nu^{(j)}$;

  Set $\beta^{(j)'} = \exp\left(f^{(j)'}\right)$;

Accept $\beta^{(1)'}, \dots, \beta^{(p)'}$ with probability given by

$$p_{\text{acc}} = \min\left(\frac{\pi\left(\mathbf{i}, \mathbf{r} | \beta^{(1)'}, \dots, \beta^{(p)'}, \lambda, \gamma, \omega, i_\omega\right)}{\pi(\mathbf{i}, \mathbf{r} | \beta^{(1)}, \dots, \beta^{(p)}, \lambda, \gamma, \omega, i_\omega)}, 1\right)$$

where δ is a tuning parameter.

The computational advantage is that the prior ratio is equivalent to the inverse of the proposal ratio and hence the probability of acceptance only depends on the augmented likelihoods ratio.

Updating the correlation parameters $\boldsymbol{\rho} = \{\rho_{j,k}\}$: We update each of the $p(p-1)/2$ correlation parameters, one at a time, using a random-walk Metropolis algorithm. That is, for a given pair of types j and k, we propose a new correlation value, $\rho'_{j,k}$, by $\rho'_{j,k} \sim N\left(\rho_{j,k}, \sigma_\rho^2\right)$, where σ_ρ^2 is a tuning parameter that can vary with j and k if necessary.

Denote by Σ' the matrix Σ constructed using $\rho'_{j,k}$ for the particular pair j and k that is being updated. If $\rho'_{j,k} \in (-1, 1)$ and Σ' is positive definite, then the proposed value is accepted with probability given by:

$$p_{\text{acc}} = \min\left(\frac{\mathcal{GP}(\log\beta; 0, \Sigma')}{\mathcal{GP}(\log\beta; 0, \Sigma)}, 1\right),$$

otherwise, $\rho'_{j,k}$ is rejected.

Updating the length scale parameter l: We update the (common across the p types) length scale parameter l using a random-walk Metropolis algorithm.

Step 3 of Algorithm 1 for the MOC Model

Propose $l' \sim N(l, \sigma^2)$;

Accept l' with probability:

$$p_{\text{acc}} = \min\left(\frac{\mathcal{GP}(\log\beta; 0, \Sigma')}{\mathcal{GP}(\log\beta; 0, \Sigma)}, 1\right),$$

where Σ' denotes the covariance matrix in (1) where each of the covariance matrices $\Sigma^{(i,j)}$, for $i, j = 1, \dots, p$, is constructed using l' and σ^2 is a tuning parameter.

B. Updating Parameters Specific to the IGP Model. For the IGP model we set $\rho_{j,k} = 0$ for all j and k and allow the covariance functions to have different length scale parameters, $l_1, \dots, l_p$. We assign independent Exponential prior distributions to l_j, $j = 1, \dots, p$ with mean $1/\chi_{l_j}$. Hence, the target posterior density up to proportionality is given by:

$$\begin{aligned} \pi(\beta, l, \gamma, \mathbf{i}, \omega, i_\omega | \mathbf{r}, \lambda) &\propto \pi(\mathbf{i}, \mathbf{r} | \beta, \lambda, \gamma, \omega, i_\omega)\, \pi(\beta | l)\, \pi(l)\, \pi(\gamma)\, \pi(\omega)\, \pi(i_\omega | \omega) \\ &\propto \prod_{j=1}^{n} h(r_j - i_j | \lambda, \gamma) \prod_{\substack{j=1 \\ j \neq \omega}}^{n} \left(\sum_{k \in \mathcal{Y}_j} \beta^{(c_j)}(x_{k,j})\right) \exp\left\{-\sum_{j=1}^{n}\sum_{k=1}^{N} \beta^{(c_k)}(x_{j,k})\delta_{j,k}\right\} \\ &\times \prod_{j=1}^{p} \mathcal{GP}\left(\log\beta^{(c_j)}; 0, \Sigma^{(j)}\right) \exp\{\chi_\omega \omega\} \exp\{-\chi_\gamma \gamma\} \prod_{j=1}^{p} \exp\{\chi_{l_j} l_j\}. \end{aligned}$$

Updating the infection rate functions $\beta = (\beta^{(1)}, \dots, \beta^{(p)})$: We update the infection rate functions for each type, one at a time, using the same proposal mechanism as in the MOC model.

Step 2 of Algorithm 1 for the IGP Model

for $j = 1, \dots p$ **do**
  Draw $\nu^{(j)} \sim \mathcal{GP}\left(0, \Sigma^{(j)}\right)$;
  Propose a new function $f^{(j)'} = \sqrt{1-\delta^2} f^{(j)} + \delta \nu^{(j)}$;
  Set $\beta^{(j)'} = \exp\left(f^{(j)'}\right)$;
  Accept $\beta^{(j)'}$ with probability given by

$$p_{\mathrm{acc}} = \min\left(\frac{\pi(\mathbf{i}, \mathbf{r} | \beta^{(1)}, \dots, \beta^{(j-1)}, \beta^{(j)'}, \beta^{(j+1)}, \dots, \beta^{(p)}, \lambda, \gamma, \omega, i_\omega)}{\pi(\mathbf{i}, \mathbf{r} | \beta^{(1)}, \dots, \beta^{(p)}, \lambda, \gamma, \omega, i_\omega)}, 1\right),$$

where $\delta \in (0, 1]$ is a tuning parameter that can vary with j if necessary.

Updating the length scale parameters $l_1, \dots, l_p$: We update the length scale parameters, one at a time, using a random-walk Metropolis algorithm.

Step 3 of Algorithm 1 for the IGP Model

for $j = 1, \dots p$ **do**
  Propose $l_j' \sim N(l_j, \sigma^2)$;
  Accept l_j' with probability given by:

$$p_{\mathrm{acc}} = \min\left(\frac{\mathcal{GP}\left(f^{(j)}; 0, \Sigma^{(j)'}\right)}{\mathcal{GP}\left(f^{(j)}; 0, \Sigma^{(j)}\right)}, 1\right),$$

where $\Sigma^{(j)'}$ denotes the covariance matrix for type j evaluated with length scale parameter l_j', and σ^2 is a tuning parameter.

C. Updating Parameters Specific to the DB Model. In the this model we first set $f^{(1)}$ as a baseline, to which we assign a GP prior with mean zero and covariance matrix $\Sigma^{(1)}$. For $j = 2, \dots p$ we then assume that

$$f^{(j)} = f^{(1)} + u^{(j)}, \quad u^{(j)} \sim \mathcal{GP}\left(0, \Sigma^{(j)}\right),$$

where $u^{(j)}$ represents the discrepancy between $f^{(j)}$ with $f^{(1)}, u^{(2)}, \dots, u^{(p)}$ assumed to be mutually independent. We note that the choice of the baseline is arbitrary due the labeling of the types and one may choose a particular type to be the basiline that aids interpretation of the results.We further assume that covariance matrices of the particular discrepancies have individual length scale parameters, $l_1, \dots, l_p$. The target posterior density up to proportionality is given by:

$$\begin{aligned}
\pi(\beta, l_1, \dots, l_p, \gamma, \mathbf{i}, \omega, i_\omega | \mathbf{r}, \lambda) \quad &\propto \quad \pi(\mathbf{i}, \mathbf{r} | \beta, l_1, \dots, l_p, \gamma, \omega, i_\omega)\, \pi(\beta | l_1, \dots, l_p)\, \pi(l_1, \dots, l_p)\, \pi(\gamma)\, \pi(\omega)\, \pi(i_\omega | \omega) \\
&\propto \quad \prod_{j=1}^{n} h(r_j - i_j | \lambda, \gamma) \prod_{\substack{j=1 \\ j \neq \omega}}^{n} \left(\sum_{k \in \mathcal{Y}_j} \beta^{(c_j)}(x_{k,j})\right) \exp\left\{-\sum_{j=1}^{n}\sum_{k=1}^{N} \beta^{(c_k)}(x_{j,k})\delta_{j,k}\right\} \\
&\times \quad \mathcal{GP}\left(\log \beta^{(1)}; 0, \Sigma^{(1)}\right) \prod_{j=2}^{p} \mathcal{GP}\left(u^{(j)}; 0, \Sigma^{(j)}\right) \exp\{\chi_\omega \omega\} \exp\{-\chi_\gamma \gamma\} \prod_{j=1}^{p} \exp\{\chi_{l_j} l_j\}.
\end{aligned}$$

Updating the infection rate functions $\beta = (\beta^{(1)}, \dots, \beta^{(p)})$: We update the infection rate functions as a block as follows:

Step 2 of Algorithm 1 for the DB Model

Draw $\nu^{(1)} \sim \mathcal{GP}\left(0, \Sigma^{(1)}\right)$;
Propose new function $f^{(1)'} = \sqrt{1-\delta^2} f^{(1)} + \delta \nu^{(1)}$;
Set $\beta^{(1)'} = \exp\left(f^{(1)'}\right)$;
for $j = 2, \dots p$ **do**
  Draw $\nu^{(j)} \sim \mathcal{GP}\left(0, \Sigma^{(j)}\right)$;
  Propose new function $u^{(j)'} = \sqrt{1-\delta^2} u^{(j)} + \delta \nu^{(j)}$;
  Set $f^{(j)'} = f^{(j-1)'} + u^{(j)'}$;
  Set $\beta^{(j)'} = \exp\left(f^{(j)'}\right)$;
Accept $\beta^{(1)'}, \dots, \beta^{(p)'}$ with probability given by $p_{\mathrm{acc}} = \min\left(\frac{\pi\left(\mathbf{i}, \mathbf{r} | \beta^{(1)'}, \dots, \beta^{(p)'}, \lambda, \gamma, \omega, i_\omega\right)}{\pi(\mathbf{i}, \mathbf{r} | \beta^{(1)}, \dots, \beta^{(p)}, \lambda, \gamma, \omega, i_\omega)}, 1\right)$.

Updating the length scale parameters $l_1, \ldots, l_p$: We update the length scale parameters, one at a time, using a random-walk Metropolis algorithm.

Step 3 of Algorithm 1 for the DB Model

---

for $j = 1, \ldots p$ **do**
  Propose $l'_j \sim N(l_j, \sigma^2)$;
  Accept l'_j with probability given by:

$$p_{\text{acc}} = \min\left(\frac{\mathcal{GP}\left(f^{(j)}; 0, \Sigma^{(j)'}\right)}{\mathcal{GP}\left(f^{(j)}; 0, \Sigma^{(j)}\right)}, 1\right),$$

where $\Sigma^{(j)'}$ denotes the covariance matrix for type j evaluated with length scale parameter l'_j, and σ^2 is a tuning parameter.

D. Updating the infectious period distribution rate parameter. Step 4 in Algorithm 1 is the same irrespective of the nonparametric model used for the infection rate function (MOC, IGP, DB). Having placed an Exponential prior distribution on γ, the full conditional distribution of γ is a Gamma distribution with shape parameter $1 + n\lambda$ and rate $\sum_{j=1}^{n}(r_j - i_j)$:

$$\gamma | \mathbf{i}, \mathbf{r}, \lambda \sim \Gamma\left(1 + n\lambda,\ \chi_\gamma + \sum_{j=1}^{n}(r_j - i_j)\right)$$

which can be sampled directly.

E. Updating the Infection Times. We treat the unobserved infection times as unknown parameters within a data-augmentation framework (2). We start by proposing to update the label of the initial infective and the corresponding initial infection times as follows:

Step 6 of Algorithm 1

Sample ω' uniformly at random from $\{1, \ldots, n\}$;
Sample $t \sim \Gamma(\lambda, \gamma)$;
Propose $i'_{\omega'} = r_{\omega'} - t$;
Accept $(\omega', i'_{\omega'})$ with probability:

$$p_{\text{acc}} = \min\left(\frac{\exp\{\chi_\omega i'_\omega\}}{\exp\{\chi_\omega i_\omega\}} \frac{h\left(r_{\omega'} - i'_{\omega'} | \lambda, \gamma\right)}{h(r_\omega - i_\omega | \lambda, \gamma)} \frac{\pi(\mathbf{i}, \mathbf{r} | \beta^{(1)}, \ldots, \beta^{(p)}, \lambda, \gamma, \omega', i'_{\omega'})}{\pi(\mathbf{i}, \mathbf{r} | \beta^{(1)}, \ldots, \beta^{(p)}, \lambda, \gamma, \omega, i_\omega)}, 1\right).$$

Updating the remaining infection times We write $\mathbf{i} + i_j$ to denote the set of infection times with i_j included and $\mathbf{i} - i_j$ to be the set with i_j removed, for some $j \in \{1, \ldots, n\}$.

Step 7 of Algorithm 1

Choose an individual, say j, uniformly at random from $\{1, \ldots, n\}$;
Sample $t_j \sim \Gamma(\lambda, \gamma)$;
Propose $i'_j = r_j - t_j$;
Accept i'_j with probability given by:

$$p_{acc} = \min\left(\frac{h(r_j - i_j | \lambda, \gamma)}{h(r_j - i'_j | \lambda, \gamma)} \frac{\pi(\mathbf{i} - i_j + i'_j, \mathbf{r} | \beta^{(1)}, \ldots, \beta^{(p)}, \lambda, \gamma, \omega, i_\omega)}{\pi(\mathbf{i}, \mathbf{r} | \beta^{(1)}, \ldots, \beta^{(p)}, \lambda, \gamma, \omega, i_\omega)}, 1\right),$$

where $\mathbf{i} - i_j + i'_j$ is the set $\mathbf{i}$ with the i_j removed and i'_j included.

One can choose, in principle, to update more than one, or even all of the infection times, one at a time sequentially (i.e. repeat Step 7 several times). However, it has been demostrated through extensive simulation studies (3) that when all the infection times are updated, although the resulting MCMC algorithm can be more efficient than when only one infection is, there is however a substantial computational cost due to repeated likelihood calculations, especially for large population size N. Therefore, there is a trade off between efficiency and computational cost.

2. Mean Projection Approximation

We use the Mean Projection Approximation (MPA) to reduce the cost of repeatedly evaluating the inverse of covariance matrices which are needed when evaluating the GP prior densities. The MPA, essentially, places a GP prior distribution on a function over a pseudo data set and then projects it onto the full data set.

Denote the function of interest over the data set d by g and the function over the pseudo data set $\bar{d}$ by $\bar{g}$. Recall the idea of the Projected Process Approximation (PPA) (see, e.g., 4), in which a joint GP prior distribution is placed on g and $\bar{g}$ such that

$$\begin{pmatrix} g \\ \bar{g} \end{pmatrix} \sim \mathcal{GP}\left(\begin{pmatrix} 0 \\ 0 \end{pmatrix}, \begin{pmatrix} \Sigma_{d,d} & \Sigma_{d,\bar{d}} \\ \Sigma_{\bar{d},d} & \Sigma_{\bar{d},\bar{d}} \end{pmatrix} \right).$$

Here, $\Sigma_{d,d}$ is a $B \times B$ matrix whose elements are the covariances between the values of the function g at two input values from the vector d, the elements of the matrix $\Sigma_{\bar{d},\bar{d}}$ of size $b \times b$ are the covariances between the values of the function $\bar{g}$ at the two input values from the vector $\bar{d}$, and $\Sigma_{d,\bar{d}}$ is a $B \times b$ matrix whose elements are the covariances between the values of the function $\bar{g}$ at input values from vector d and the values of the function $\bar{g}$ at input values from the vector $\bar{d}$. It follows that the conditional distribution $g|\bar{g}$ is $\mathcal{GP}\left(\Sigma_{d,\bar{d}}\Sigma_{\bar{d},\bar{d}}^{-1}\bar{g},\ \Sigma_{d,d} - \Sigma_{d,\bar{d}}\Sigma_{\bar{d},\bar{d}}^{-1}\Sigma_{\bar{d},d} \right)$.

Adapting the idea of PPA to our setting, the MPA projects the function $\bar{g}$ over the pseudo dataset $\bar{d}$ onto the full data set d by using the *mean* of the conditional distribution of $g|\bar{g}$:

$$g = \Sigma_{d,\bar{d}}\Sigma_{\bar{d},\bar{d}}^{-1}\bar{g}. \qquad [2]$$

In other words, the values of g over the full data set are deterministic functions of the values of $\bar{g}$ over the pseudo data set. In particular, we can obtain g using the inverse of $\Sigma_{\bar{d},\bar{d}}$ rather than the inverse of $\Sigma_{d,d}$, which would have been computationally prohibitive.

Although some care is required to construct the pseudo set $\bar{d}$ to ensure that the points are sufficient in number and suitably placed across the entire domain to capture the features of the function f, simulation studies in (5) suggest that the error introduced by MPA is small, even when the size of $\bar{d}$ is relatively small, e.g. 10% of d.

A. Implementing MPA for the MOC model. We demonstrate how to sample from the target posterior density of the MOC model when the MPA is employed. We place the joint prior distribution of (2) on the functions $f^{(j)}$ and $\bar{f}^{(j)}$ for each type $j = 1, \ldots, p$. We further assume that the joint distribution of $f^{(j)}$ and $\bar{f}^{(j)}$ is independent of the joint distribution of $f^{(m)}$ and $\bar{f}^{(m)}$ for any $m \neq j$, $m = 1, \ldots, p$. As mentioned above, the MPA exploits the conditional distribution of $f^{(j)}|\bar{f}^{(j)}$ and in particular each $\bar{f}^{(j)}$ is projected to $f^{(j)}$ using its mean and this is done for the function of each type, independently of each other.

Updating the infection rate functions $\beta = \left(\beta^{(1)}, \ldots, \beta^{(p)}\right)$: We modify Step 2 of Algorithm 1 to sample from the infection rate functions $\beta^{(1)}, \beta^{(2)}, \ldots, \beta^{(p)}$ as follows:

Step 2 of Algorithm 1 for the MOC Model using MPA

Draw $\bar{\nu}^{(1)}, \ldots, \bar{\nu}^{(p)}$ from the joint GP prior distribution on the input space $\bar{d}$:

$$\begin{pmatrix} \bar{\nu}^{(1)} \\ \bar{\nu}^{(2)} \\ \vdots \\ \bar{\nu}^{(p)} \end{pmatrix} \sim \mathcal{GP}\left(0, \begin{pmatrix} \Sigma^{(1,1)} & \cdots & \rho_{1,p}\Sigma^{(1,p)} \\ \rho_{2,1}\Sigma^{(2,1)} & \cdots & \rho_{2,p}\Sigma^{(2,p)} \\ \vdots & & \vdots \\ \rho_{p,1}\Sigma^{(p,1)} & \cdots & \Sigma^{(p,p)} \end{pmatrix} \right);$$

for $j = 1, \ldots p$ **do**

  Propose a new function $\bar{f}^{(j)'} = \sqrt{1-\delta^2}\bar{f}^{(j)} + \delta\bar{\nu}^{(j)}$;

  Project $\bar{f}^{(j)'}$ onto $f^{(j)'}$ using (2), i.e. $f^{(j)'} = \Sigma_{d,\bar{d}}\Sigma_{\bar{d},\bar{d}}^{-1}\bar{f}^{(j)'}$;

  Set $\beta^{(j)'} = \exp\left(f^{(j)'}\right)$;

Accept $\beta^{(1)'}, \ldots, \beta^{(p)'}$ with probability given by

$$p_{\text{acc}} = \min\left(\frac{\pi\left(\mathbf{i}, \mathbf{r}|\beta^{(1)'}, \ldots, \beta^{(p)'}, \lambda, \gamma, \omega, i_\omega\right)}{\pi(\mathbf{i}, \mathbf{r}|\beta^{(1)}, \ldots, \beta^{(p)}, \lambda, \gamma, \omega, i_\omega)}, 1 \right)$$

where δ is a tuning parameter.

Updating the correlation parameters $\boldsymbol{\rho} = \{\rho_{j,k}\}$: We update each of the $p(p-1)/2$ correlation parameters, one at a time, using a random-walk Metropolis algorithm. That is, for a given pair of types j and k, we propose a new correlation value, $\rho'_{j,k}$, by $\rho'_{j,k} \sim N\left(\rho_{j,k}, \sigma_\rho^2\right)$, where σ_ρ^2 is a tuning parameter that can vary with j and k if necessary.

Denote by $\bar{\Sigma}'$ the matrix Σ in (1) constructed on the input space $\bar{d}$ and using $\rho'_{j,k}$ for the particular pair j and k that is being updated. If $\rho'_{j,k} \in (-1,1)$ and $\bar{\Sigma}'$ is positive definite, then the proposed value is accepted with probability given by:

$$p_{\text{acc}} = \min\left(\frac{\mathcal{GP}(\log\beta; 0, \bar{\Sigma}')}{\mathcal{GP}(\log\beta; 0, \bar{\Sigma})}, 1\right),$$

otherwise, $\rho'_{j,k}$ is rejected.

Updating the length scale parameter l: We update the (common across the p types) length scale parameter l using a random-walk Metropolis algorithm.

Step 3 of Algorithm 1 for the MOC Model

1: Propose $l' \sim N(l, \sigma^2)$;
2: Accept l' with probability:

$$p_{\text{acc}} = \min\left(\frac{\mathcal{GP}(\log\beta; 0, \bar{\Sigma}')}{\mathcal{GP}(\log\beta; 0, \bar{\Sigma})}, 1\right),$$

where $\bar{\Sigma}'$ denotes the covariance matrix in (1) constructed on the input space $\bar{d}$ and with each of the covariance matrices $\Sigma^{(i,j)}$, for $i, j = 1, \ldots, p$, constructed using l' and σ^2 is a tuning parameter.

3. Synthetic Data for Two Types: Numerical Comparison

We present here a numerical comparison for the simulation study with synthetic data for two types. We define the maximum absolute error for farm type m by

$$\text{AE}^{(m)} = \max_{j,k} |\beta^{(m)}_{jk} - \hat{\beta}^{(m)}_{jk}|$$

where $\hat{\beta}^{(m)}_{jk}$ is the posterior median of the model in question. These values, given in Table S1, show that the MOC model performs best for the type 0 infection rate, and both the MOC and IGP models perform well for the type 1 infection rate.

Table S1. Maximum absolute error for the estimates for type zero and type one infection rates.

Model	Error for Type 0 ($\times 10^{-4}$)	Error for Type 1 ($\times 10^{-4}$)
IGP	5.4	1.04
MOC	1.86	1.32
DB	6.94	2.70

4. Assessing Disease Control Strategies

A. Simulation Study I. We chose 1,000 farms uniformly at random from the 2001 UK Foot and Mouth disease data set described in the main text, and simulated an outbreak assuming that all farms were of the same type and with infection rate

$$\tilde{\beta}_{ij} = \beta_{ij}(d_{ij}) = 0.3 \times 0.0015\left(1 + (d_{ij} - 2)^2\right)^{-1} + 0.7 \times 0.0015\left(1 + d_{ij}^2\right)^{-1},$$

where d_{ij} denotes the Euclidean distance between farms i and j. We further assumed a Gamma distributed infectious period with mean 6 days and standard deviation 3.46 days. We fitted six models to the data set consisting of the simulated infection and removal times, with the only difference between the models being the assumption about the functional form of the infection kernel (see Table 2 in the main text). As there is only one type of farm in the data set, the nonparametric models described in the main text (IGP, MOC and DB) are identical and denoted by M_6 in Table 2 in the main text. We fitted M_6 with fixed hyperparameters $\alpha = 6$ and $l = 3$km. In addition, we fitted five parametric models denoted by $M_1, \ldots, M_5$, with their model parameters being inferred from the data assuming vague, independent Exponential prior distributions with rate 0.01. We further assumed a Gamma infectious period distribution with shape parameter fixed as $\lambda = 3$ and inferred the rate parameter γ, also placing a vague, Exponential prior distribution on γ with rate 0.01.

Figure S1 shows the fitted parametric functions using the posterior mean parameter values, the posterior mean of our Bayesian nonparametric function, and the true mixture function. Model M_2 overestimates the long-range infection rates and underestimates short-range infection rates. Model M_3 has an exponential form and severely underestimates the infection rate for the first 5km by having to allow for the very long range transmissions in the data. Models M_4 and M_5 fail to infer the infection rate over short distances due to the mixture dynamics of the true model. Only the Bayesian nonparametric model, M_6, correctly infers the mixture structure.

For each of the five models, having obtained samples from the posterior distribution of the infection rate function, we drew 1,000 samples from the posterior predictive distribution of the infection and removal times. This was done by simulating outbreaks where the infection rate function was a draw from its posterior distribution. This then gave rise to the posterior predictive distribution of the final size, i.e. the number of initially susceptible farms that became infected at some time during the outbreak.

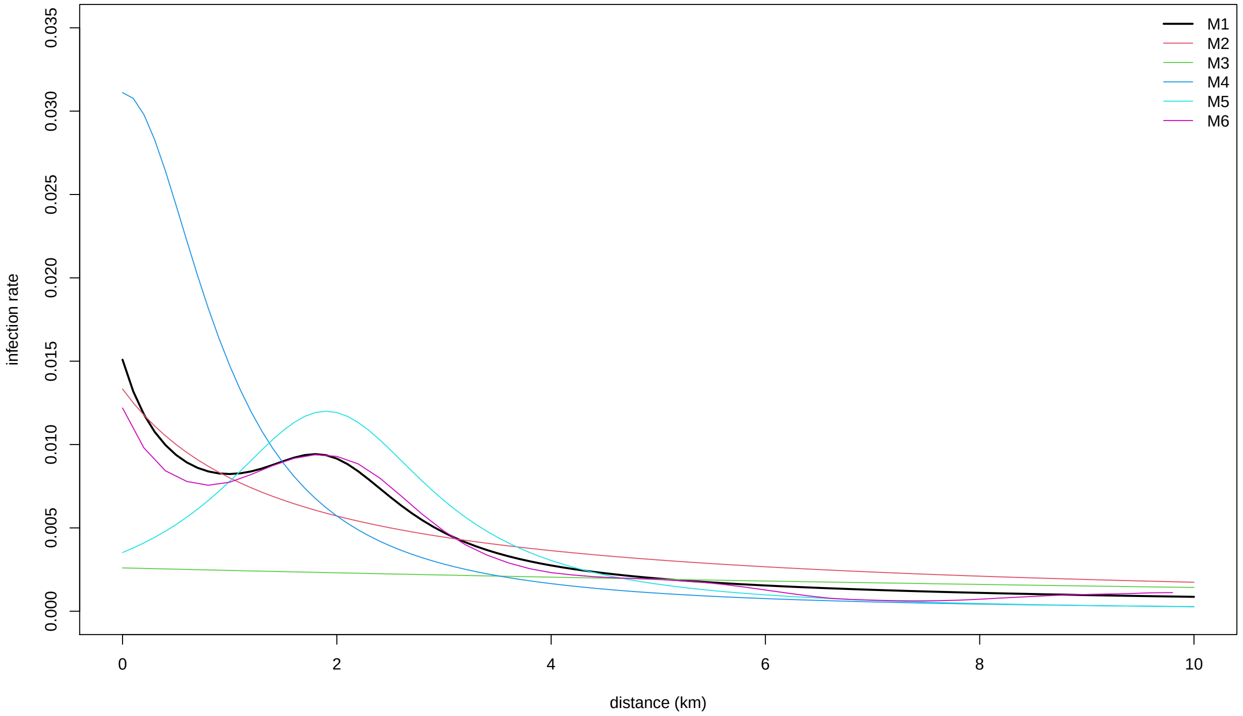

Fig. S1. The fitted functions for Simulation Study I. The parametric functions use the posterior mean estimates for their parameter values, and the Bayesian nonparametric using the posterior mean of the GP. Model M_1 is the fitted parametric version of the true model, M_6 is the GP model and models M_2 - M_5 are alternative parametric models.

To investigate the effect of a ring-culling strategy as part of a control measure, we followed (6) and again simulated outbreaks from the posterior predictive distribution of the infection and removal times. In contrast to the method described above, this time all farms within 3 km were also removed/culled, regardless of their infection status. To mimic a realistic response, we assumed the authorities only began culling when 30 farms are infected, and before 60 farms were infected it was assumed that the available resources were only sufficient to cull at a 1.5 km radius. Table 2 in the main text shows the results which reveal that only our nonparametric model, M_6, closely matches both the predicted mean final size and probability of a severe outbreak (i.e. one in which at least 10% of farms were infected) with the values predicted from the true model M_1.

B. Simulation Study II. We carried out a second simulation study to assess the ability of our method to estimate the probability of a severe outbreak and the final size, the former again defined as an epidemic where at least 10% of the initially susceptible farms were infected. We chose 1,000 farms uniformly at random from the 2001 UK FMD disease data, and simulated an outbreak assuming that all farms were of the same type and infection rate $\tilde{\beta}_{ij} = \beta_{ij}(d_{ij}) = 0.7\exp\{-0.7d_{ij}\}$, where d_{ij} denotes the Euclidean distance between farms i and j. Infectious periods were assumed to be Gamma distributed with mean 6 days and standard deviation 3.46 days. The simulated outbreak lasted 66 days and 665 farms were infected. We considered three models, the only difference between them being the assumption about the functional form of the infection function (see Table S2). We fitted two parametric models denoted by M_1 and M_2, with their parameters, (θ_1, θ_2) and λ_1 respectively, being inferred from the data assuming vague, independent Exponential prior distributions with rate 0.01. Since there is only one type of farm in the dataset, the nonparametric models described in the main text (IGP, MOC and DB) are identical and denoted by M_3 in Table S2. We fitted M_3 with fixed hyperparameters $\alpha = 6$ and $l = 4$km. We also assumed a Gamma infectious period distribution with shape parameter fixed as $\lambda = 3$ and inferred the rate parameter γ, also placing a vague, Exponential prior distribution on γ with rate 0.01.

Table S2. The results of the ring-culling strategy including the time taken to run the MCMC algorithm.

Model	Infection function (β_{ij})	Mean final size	Probability of a severe outbreak	Time taken (mins)
M_1 (Exponential)	$\theta_1\exp\{-\theta_2 d_{ij}\}$	327	0.790	2
M_2 (Logistic)	$\lambda_1/(1+d_{ij})$	555	0.658	0.167
M_3 (GP)	$\exp(f(d_{ij}))$	303	0.796	60

We also investigated the effect of a ring-culling strategy as a control measure by simulating outbreaks from the posterior predictive distribution of the infection and removal times. We make the same assumptions about the culling procedures as in Simulation Study I described above. Table S2 shows that under the true model M_1 the culling strategy results in the final size being roughly halved, from 665 to 327, and the probability of a serious outbreak, where more than 10% of farms are infected, is 0.790. Model M_2 leads to erroneous conclusions, falsely showing that culling only reduces the number of infected farms by 17% and suggesting a reduced probability of a severe outbreak. Conversely, our nonparametric approach matches the results from the true model M_1 both in terms of final size and the probability of a severe outbreak, and does so without having to specify the parametric form of the infection rate function.

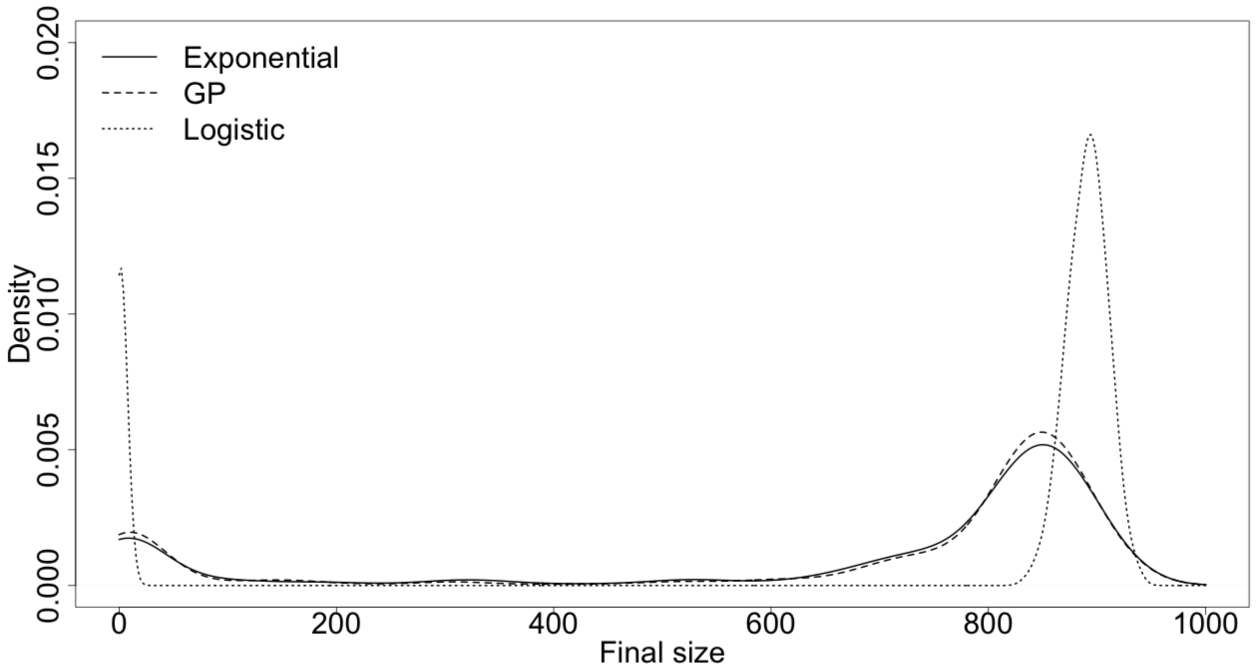

Fig. S2. Predictive final size distribution for parametric and nonparametric models. The exponential model, M_1, is the true model, the logistic model is M_2 and the GP is M_3.

References

1. R Neal, Regression and classification using Gaussian process priors, in *Bayesian Statistics 6*, eds. JM Bernardo, JO Berger, AP Dawid, AFM Smith. (Oxford Univeristy Press), (1998).
2. PD O'Neill, GO Roberts, Bayesian inference for partially observed stochastic epidemics. *J Roy Stat Soc A* **162**, 121–129 (1999).
3. T Kypraios, *Efficient Bayesian Inference for Partially Observed Stochastic Epidemics and A New Class of SemiParametric Time Series Models.* (Lancaster University), (2007).
4. CE Rasmussen, CKI Williams, *Gaussian Processes for Machine Learning.* (The MIT Press), (2005).
5. RG Seymour, Bayesian nonparametric methods for individual-level stochastic epidemic models. *University of Nottingham* (2020).
6. RG Seymour, T Kypraios, PD O'Neill, A Bayesian nonparametric analysis of the 2003 outbreak of highly pathogenic avian influenza in the Netherlands. *J. Royal Stat. Soc. Ser. C* **70** (2021).
